# Effects of hydrogen sulfide on hemodynamics, inflammatory response and oxidative stress during resuscitated hemorrhagic shock in rats

**DOI:** 10.1186/cc9257

**Published:** 2010-09-13

**Authors:** Frédérique Ganster, Mélanie Burban, Mathilde de la Bourdonnaye, Lionel Fizanne, Olivier Douay, Laurent Loufrani, Alain Mercat, Paul Calès, Peter Radermacher, Daniel Henrion, Pierre Asfar, Ferhat Meziani

**Affiliations:** 1 Laboratoire HIFIH, UPRES EA 3859, IFR 132, Université d'Angers, Rue Haute de Reculée, Angers, F-49035 France; 2 Département de Réanimation Médicale et de Médecine Hyperbare, Centre Hospitalo- Universitaire, 4 rue Larrey, Angers, F-49035, France; 3 INSERM UMR 771; CNRS UMR 6214; Université d'Angers, Rue Haute de Reculée, Angers, F-49035, France; 4 Sektion Anästhesiologische Pathophysiologie und Verfahrensentwicklung, Klinik für Anästhesiologie, Universitätsklinikum, Parkstrasse 11, Ulm, D-89073, Germany; 5 Laboratoire de Biophotonique et Pharmacologie, UMR 7213 CNRS, Université de Strasbourg, Faculté de Pharmacie, 74 route du Rhin, Illkirch, F-67401, France; 6 Service de Réanimation Médicale, Nouvel Hôpital Civil. Hôpitaux Universitaires de Strasbourg. 1, place de l'Hôpital, F-67031 Strasbourg, France

## Abstract

**Introduction:**

Hydrogen sulfide (H_2_S) has been shown to improve survival in rodent models of lethal hemorrhage. Conversely, other authors have reported that inhibition of endogenous H_2_S production improves hemodynamics and reduces organ injury after hemorrhagic shock. Since all of these data originate from unresuscitated models and/or the use of a pre-treatment design, we therefore tested the hypothesis that the H_2_S donor, sodium hydrosulfide (NaHS), may improve hemodynamics in resuscitated hemorrhagic shock and attenuate oxidative and nitrosative stresses.

**Methods:**

Thirty-two rats were mechanically ventilated and instrumented to measure mean arterial pressure (MAP) and carotid blood flow (CBF). Animals were bled during 60 minutes in order to maintain MAP at 40 ± 2 mm Hg. Ten minutes prior to retransfusion of shed blood, rats randomly received either an intravenous bolus of NaHS (0.2 mg/kg) or vehicle (0.9% NaCl). At the end of the experiment (T = 300 minutes), blood, aorta and heart were harvested for Western blot (inductible Nitric Oxyde Synthase (iNOS), Nuclear factor-κB (NF-κB), phosphorylated Inhibitor κB (P-IκB), Inter-Cellular Adhesion Molecule (I-CAM), Heme oxygenase 1(HO-1), Heme oxygenase 2(HO-2), as well as nuclear respiratory factor 2 (Nrf2)). Nitric oxide (NO) and superoxide anion (O_2_^-^) were also measured by electron paramagnetic resonance.

**Results:**

At the end of the experiment, control rats exhibited a decrease in MAP which was attenuated by NaHS (65 ± 32 versus 101 ± 17 mmHg, *P *< 0.05). CBF was better maintained in NaHS-treated rats (1.9 ± 1.6 versus 4.4 ± 1.9 ml/minute *P *< 0.05). NaHS significantly limited shock-induced metabolic acidosis. NaHS also prevented iNOS expression and NO production in the heart and aorta while significantly reducing NF-kB, P-IκB and I-CAM in the aorta. Compared to the control group, NaHS significantly increased Nrf2, HO-1 and HO-2 and limited O_2_^- ^release in both aorta and heart (*P *< 0.05).

**Conclusions:**

NaHS is protective against the effects of ischemia reperfusion induced by controlled hemorrhage in rats. NaHS also improves hemodynamics in the early resuscitation phase after hemorrhagic shock, most likely as a result of attenuated oxidative stress. The use of NaHS hence appears promising in limiting the consequences of ischemia reperfusion (IR).

## Introduction

Hemorrhagic shock (HS) is a life-threatening complication in both trauma patients and in the operating room [1,2]. The pathophysiology of HS is complex, especially during the reperfusion phase [3]. During HS, the state of vasoconstriction turns into vasodilatory shock. According to Landry *et al. *[4], this phenomenon is related to tissue hypoxia as well as to a proinflammatory immune response [4]. In addition, during the reperfusion phase, cellular injuries induced by ischemia are enhanced, and are associated with excessive production of radical oxygen species (ROS), leading to a further systemic inflammatory response [5].

Hydrogen sulfide (H_2_S), is known as an environmental toxic gas [6], but has also recently been recognized as a gasotransmitter [7], similar to nitric oxide (NO) and carbon monoxide (CO). H_2_S is endogenously synthesized [8] and may play a crucial role in critical care according to the recent review of Wagner *et al. *in 2009 [9]. Depending on the selected models, H_2_S has been reported to exhibit pro- and anti-inflammatory properties and to display opposite effects in various shock conditions [10-13]. H_2_S has also been reported to induce direct inhibition of endothelial nitric oxide synthase (eNOS) [14]. However, this effect was linked to the concentration of H_2_S, whereby H_2_S caused contraction at low doses and relaxation at high doses in both rat and mouse aorta precontracted by phenylephrine [14]. This dual effect was related, at low dosage, to the inhibition of the conversion of citrulline into arginine by eNOS (contraction) and at high dosage by activation of K^+^_ATP _channels or due to NO quenching [15]. Blackstone *et al. *[10,11] recently suggested that inhalation of H_2_S induced a "suspended animation-like" state which protected animals from lethal hypoxia. Furthermore, Morrison *et al. *[16] demonstrated that pre-treatment with inhaled or intravenous (*i.v*.) H_2_S prevented death and lethal hypoxia in rats subjected to controlled but unresuscitated hemorrhage.

Conversely, Mok *et al. *[17] reported the hemodynamic effects of the inhibition of H_2_S synthesis, along with a rapid restoration in mean arterial pressure (MAP) and heart rate (HR), in a model of unresuscitated hemorrhage in rats.

As the vascular effects of H_2_S are still a matter of debate, and since all of these data originated from unresuscitated hemorrhage, we therefore tested the hypothesis that the H_2_S donor sodium hydrosulfide (NaHS), infused before retransfusion in a model of a controlled hemorrhagic rat, may improve hemodynamics and attenuate oxidative and nitrosative stresses, as well as the inflammatory response during reperfusion. Since the role of the cardiovascular system during shock becomes critical, we therefore focused on the inflammatory response as well as on the oxidative and nitrosative stresses in the heart and aorta.

## Materials and methods

The animal protocol was approved by the regional animal ethics committee (CREEA-Nantes, France). The experiments were performed in compliance with the European legislation on the use of laboratory animals.

### Animals

Adult male Wistar rats, weighing 325 ± 15 g, were housed with 12-hour light/dark cycles in the animal facility of the University of Angers (France).

### Surgical procedure

Animals were anesthetized with intraperitoneal pentobarbital (50 mg/kg of body weight) and placed on a homeothermic blanket system in order to maintain rectal temperature between 36.8°C and 37.8°C throughout the experiment. After local anesthesia with lidocaine 1% (Lidocaine^® ^1% AstraZeneca, Reuil-Malmaison, France), a tracheotomy was performed. Animals were mechanically ventilated (Harvard Rodent 683 ventilator, Harvard Instruments, South Natick, MA, USA) and oxygen was added in order to maintain PaO_2 _above 100 mmHg. The left carotid artery was exposed, and a 2.0 mm transit-time ultrasound flow probe (Transonic Systems Inc., Ithaca, NY, USA) was attached to allow continuous measurement of blood flow (CBF).

After local anesthesia, the femoral artery was canulated both to measure MAP and HR and for the induction of hemorrhagic shock. The homolateral femoral vein was canulated for retransfusion of shed blood, for fluid maintenance and for bolus infusion (either vehicle or NaHS).

### Induction of hemorrhagic shock and protocol design

**Figure 1:**
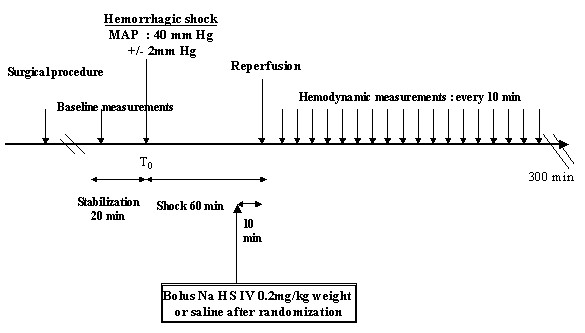
**Design of the protocol (in case of hemorrhagic shock)**.

After a 20-minute stabilization period, controlled hemorrhage [18] was induced by withdrawing approximately 9 ml of blood collected in a heparinized syringe (200 UI) within 10 minutes until MAP decreased to 40 ± 2 mmHg. This state of controlled hemorrhage was maintained during 60 minutes by further blood withdrawal or reinfusion of shed blood. Ten minutes prior to retransfusion time, rats were randomly allocated to receive either NaHS (single *i.v*. bolus 0.2 mg/kg body weight) or control (vehicle 0.9% NaCl), and designated as HS-NaHS (*n *= 11) and HS-saline (*n *= 11) respectively. After 60 minutes of shock, shed blood was retransfused within 10 minutes. Animals were continuously monitored for HR, MAP and CBF during 300 minutes. At the end of the experiment, the rats were sacrificed and blood samples were collected for measurement of arterial lactate levels. Aorta and hearts were harvested and maintained in liquid nitrogen for further *in vitro *analyses (Western blotting, superoxide anion and NO production) (Figure 1).

Two additional groups of rats were managed in the same manner as the other animals but were not bled. One group (control-NaHS, *n *= 5) received a single bolus of NaHS (0.2 mg/kg body weight) while the other group received the vehicle (0.9% NaCl 0.2 mg/kg body weight) (control-saline *n *= 5) in order to assess the hemodynamic effects of NaHS in normal rats.

Maintenance of fluid was performed with a perfusion of 1.2 ml per hour of 0.9% NaCl in all groups.

### Hydrogen sulfide donor preparation

The dehydrated NaHS powder (sodium hydrogen sulfide, anhydrous, 2 g, Alpha Aesar GmbH & Co, UK) was dissolved in isotonic saline under argon gas bubbling, until a concentration of 40 mM was achieved. Intravenous (*i.v*.) administration was preferred to the inhaled form of H_2_S, as it represented an easier route whilst avoiding side effects such as airway irritation. In accordance with pilot experimentations in our laboratory and a previous study [19], a single intravenous bolus of NaHS (0.2 mg/kg) was infused.

### Monitoring and measurements

Arterial blood gases were controlled after the stabilization period in order to adjust mechanical ventilation. Blood gases, acid-base status and blood glucose were recorded at baseline (t = 0 minute), at the end of retransfusion (t = 70 minutes) and at the end of the experiment (t = 300 minutes). MAP, HR, CBF and temperature were recorded during the stabilization period (baseline) and every 10 minutes during the observation period.

### *In vitro *measurements

#### Determination by electron paramagnetic resonance (EPR) NO spin trapping

Aorta and heart samples were incubated for 30 minutes in Krebs-Hepes buffer containing: BSA (20.5 g/L), CaCl_2 _(3 mM) and L-Arginine (0.8 mM). N, N D-Ethyldithiocarbamate and Fe^3^+ citrate complex (FeDETC) (3.6 mg) and FeSO_4_.7H_2_O (2.25 mg) were separately dissolved under N_2 _gas bubbling in 10 ml volumes of ice-cold Krebs-Hepes buffer. These compounds were rapidly mixed to obtain a pale yellow-brown opalescent colloid Fe(DETC)_2 _solution (0.4 mM), which was used immediately. The colloid Fe(DETC)_2 _solution was added to the organs and incubated for 45 minutes at 37°C. Thereafter, the organs were snap frozen in plastic tubes using liquid N_2_. NO measurement was performed on a table-top x-band spectrometer Miniscope (Magnettech, MS200, Berlin, Germany). Recordings were performed at 77°K, using a Dewar flask. Instrument settings were: microwave power, 10 mW; amplitude modulation, 1 mT; modulation frequency, 100 kHz; sweep time, 60 s and number of scans, 5. Levels of NO were expressed as amplitude of signal in unit per weight of dried sample (Amplitude/Wd).

#### Superoxide anion (O_2_^-^) spin-trapping

Aorta and heart samples were allowed to equilibrate in deferoxamine-chelated Krebs-Hepes solution containing 1 hydroxy-3methoxycarbonyl 2,2,5,5-tetramethylpyrrolidin (CMH, Noxygen, Germany) (500 μM), deferoxamine (25 μM) and DETC (5 μM) under constant temperature (37°C) for one hour. The reaction was stopped by placing the samples in ice, subsequently frozen in liquid N_2 _and analyzed in a Dewar flask by EPR spectroscopy (Magnettech, MS200, Berlin, Germany).. The instrument settings were as follows: temperature, 77° K; microwave power, 1 mW; amplitude modulation, 0.5 mT; sweep time, 60 s; field sweep, 60 G. Values were expressed in signal amplitude/mg weight of dried tissue (Amplitude/Wd).

### Western blotting

Aorta and heart samples were homogenized in lysis buffer (0.5 M Tris-HCl, 1.86 g/ml EDTA, 1 M NaCl, 0.001 g/ml Digitonin, 4 U/ml Aprotinin, 2 μM Leupeptin, 100 μM phenylmethylsulfonyl fluoride (PMSF)). Proteins (20 μg) were separated on 10% SDS-PAGE and transferred onto nitrocellulose membranes. Blots were probed by an over-night incubation (4°C) with a mouse anti-inducible NOS (iNOS) antibody (BD Biosciences, San Jose, CA, USA), a polyclonal rabbit nuclear factor NF-kB p65 antibody (Abcam, Cambridge, UK), a mouse anti-human phosphorylated (ser32/36)-IkB alpha (P-IkBa) antibody (US Biologica, Swampscott, Massachusetts, USA), an anti-rat I-CAM/CD54 antibody (R&D Systems), a goat COX-1(M-20) antibody (Santa Cruz Biotechnology, Santa Cruz, CA, USA), a goat COX-2 antibody (Santa Cruz Biotechnology), a rabbit polyclonal nuclear respiratory factor Nrf2 (C-20) antibody (Santa Cruz Biotechnology), a rabbit anti-heme-oxygenase-1 (HO-1) polyclonal antibody (Stressgen Bioreagents, San Diego California, USA) or a rabbit anti-heme-oxygenase-2 (HO-2) polyclonal antibody (Stressgen Bioreagents, San Diego California, USA). Membranes were washed and incubated for one hour at room temperature with a secondary anti-mouse, anti-rabbit or anti-goat peroxidase-conjugated IgG (Promega, Madison, WI, USA).

Blots were visualized using an enhanced chemiluminescence system (ECL Plus; Amersham, Buckinghamshire, UK), after which the membranes were probed again with a polyclonal rabbit anti-β-actin antibody (Sigma-Aldrich, Saint Quentin Fallavier, France) for densitometric quantification and normalization to β-actin expression.

### Data analysis

For repeated measurements, one-way analysis of variance was used to evaluate within-group differences. Difference between groups was tested using a two-way analysis of variance (repeated time measurements and treatments as independent variables). When the relevant F values were significant at the 5% level, further pairwise comparisons were performed using the Dunnett's test for the effect of time and with Bonferroni's correction for the effects of treatment at specific times. The Mann-Whitney test was used for inter-group comparisons for Western blotting, NO and O_2_*^- ^*signal measurements. All values are presented as mean ± SD for n experiments (n representing the number of animals). All statistics were performed with the Statview software (version 5.0; SAS Institute, Cary, NC, USA). A *P-*value < 0.05 was considered statistically significant.

## Results

### The hydrogen sulfide donor, NaHS, prevents ischemia-reperfusion (I/R)-induced hemodynamic dysfunction

**Figure 2:**
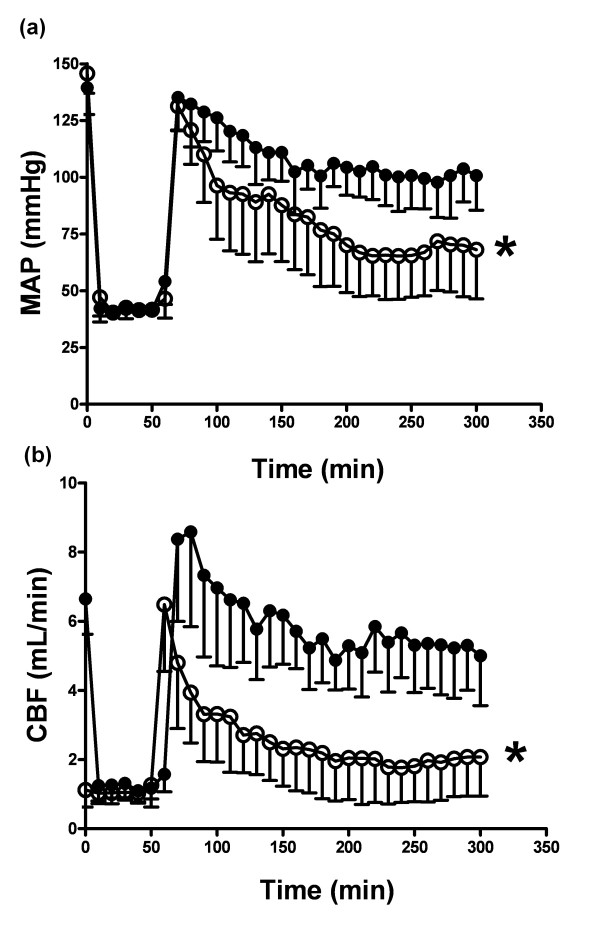
**Hemodynamic measurements**. Mean arterial blood pressure (MAP) and carotid blood flow (CBF) in hemorrhagic shock (HS)/saline group (white circle) and hemorrhagic shock/NaHS group (black circle) rats recorded during 300 minutes monitoring period. Data are expressed as mean ± SD of *n *= 11 rats for HS/NaHS group, *n *= 11 rats for HS/saline group. *P < 0.05, significantly different between HS-saline and HS-NaHS groups.

There was no significant difference in hemodynamic parameters at baseline (Table 1, Figure 2). Both hemorrhage groups were similarly bled (9.2 ± 1.8 mL versus 9.2 ± 1.6 mL for HS-saline and HS-NaHS respectively). While HR was unaffected, MAP and CBF remained significantly decreased after controlled HS despite retransfusion of shed blood, although this effect was significantly (*P *< 0.05) attenuated in HS-NaHS-treated animals (Figure 2). All HS-NaHS-treated animals survived, whereas 5 animals out of 11 died in the HS-saline group within five hours of experimentation from refractory hypotension. The mean survival time in the HS-saline group was 230 ± 89 minutes. Arterial pH and base excess were similar at baseline.

**Table 1:** Hemodynamic and acid-base measurements

	Control saline group (*n *= 5)	Control NaHS group (*n *= 5)	HS saline group (*n *= 11)	HS NaHS group (*n *= 11)
**MAP (mmHg)**				
Baseline	145 ± 8	147 ± 16	146 ± 13	140 ± 12
Reperfusion	128 ± 18	128 ± 18	131 ± 16	135 ± 14
End experiment	119 ± 20	126 ± 8	65 ± 32^§^	101 ± 16.5*^§^
**HR (beat/min)**				
Baseline	414 ± 25	402 ± 55	406 ± 45	414 ± 40
Reperfusion	408 ± 34	408 ± 34	420 ± 43	398 ± 45
End experiment	414 ± 25	426 ± 33	429 ± 45	423 ± 57
**CBF (ml/min)**				
Baseline	5.4 ± 1.1	5.5 ± 2.3	7.1 ± 2.7	7.2 ± 2.3
Reperfusion	4.8 ± 0.7	6.3 ± 3.5	6.5 ± 2.9	8.4 ± 3.5
End experiment	4.1 ± 1.4	7.1 ± 3.9	1.86 ± 1.6^§^	4.4 ± 1.9*^§^
**pH**				
Baseline	7.41 ± 0.04	7.40 ± 0.09	7.34 ± 0.05	7.35 ± 0.03
Reperfusion	7.42 ± 0.04	7.38 ± 0.08	7.23 ± 0.12	7.22 ± 0.10
End experiment	7.40 ± 0.08	7.41 ± 0.04	7.27 ± 0.11	7.34 ± 0.09*^§^
**Base excess (mM)**				
Baseline	4.56 ± 1.55	3.76 ± 1.30	2.41 ± 1.69	2.98 ± 1.71
Reperfusion	4.96 ± 2.16	2.82 ± 1.79	-7.24 ± 6.7	-3.28 ± 3.24
End experiment	2.60 ± 1.86	2.14 ± 2.74	-7.61 ± 7.23	-2.17 ± 3.58*

MAP, mean arterial pressure; HR, heart rate; CBF, carotid blood flow.

* *P *< 0.05 *vs*. HS saline. ^§^*P *< 0.05 *vs*. reperfusion.

Compared to the control group, NaHS significantly limited the decrease in pH during the reperfusion period (*P *< 0.05) (Table 1). In both control-saline and control-NaHS groups, hemodynamics remained unaltered (MAP, CBF and HR), as was arterial pH. Hence, EPR and Western blot analysis were not performed in these groups.

### NaHS prevents I/R-dependent iNOS expression and NO overproduction in cardiovascular tissues

**Figure 3:**
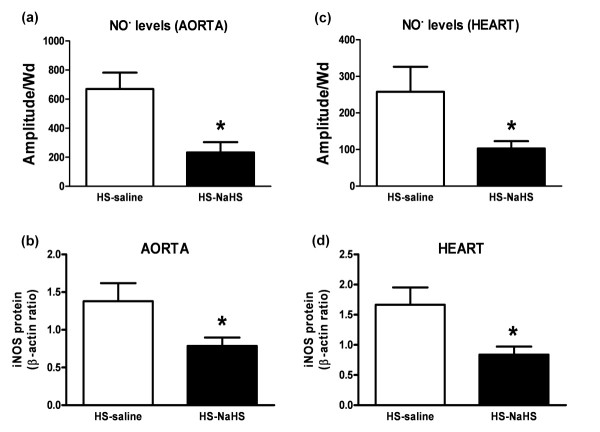
**NaHS administration reduces NO production and iNOS expression in aorta and heart**. **(a, c) **Quantification of the amplitude of NO-Fe(DETC)_2 _signal in unit/weight (mg of the dried sample Amplitude/Wd, *n *= 10) in the aorta (a) and heart (c) of the two groups of rats. **(b, d) **Western blots revealing iNOS expression in the in the whole lysate of aortas (*n *= 6) (b) and in hearts (*n *= 6) (d) of two groups of rats. Densitometric analysis was used to calculate normalized protein ratio (protein to β-actin). Data are expressed as mean ± SD. *P < 0.05, significantly different between HS-saline and HS-NaHS groups.

Compared to the HS-saline group, NaHS treatment in hemorrhagic rats prevented I/R-induced NO overproduction in the aorta and heart (*P *< 0.05) (Figure 3a, c). In agreement with these data, a decreased iNOS protein concentration was found in both aorta and heart in the HS-NaHS group (Figure 3b, d).

### NaHS reduces I/R-induced up-regulation of cardiovascular phosphorylated I-κB and cell adhesion molecules in aorta

**Figure 4:**
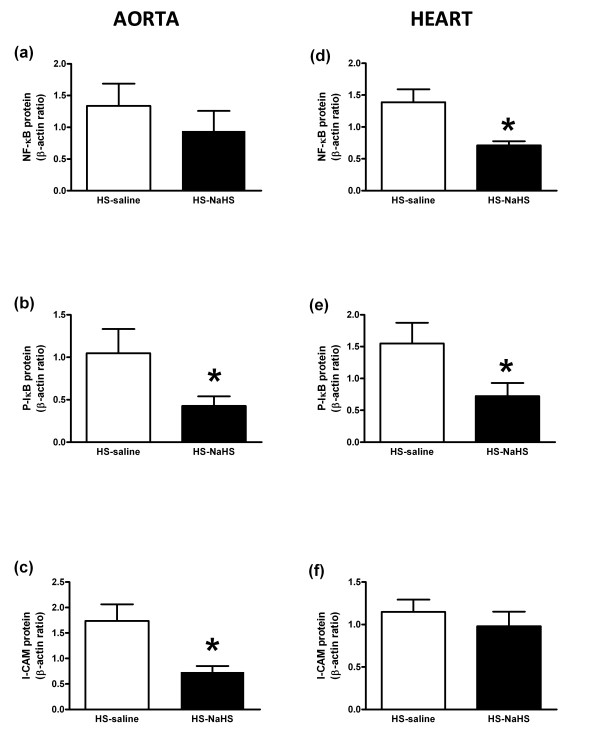
**Effects of NaHS on inflammatory pathway signaling**. **(a, d) **Western blots revealing NF-kB expression in the aorta (a) and in the heart (d). **(b, e) **Western blots revealing P-IκB expression in aorta (b) and in heart (e). **(c, f) **Western blots revealing I-CAM expression in aorta (c) and in heart (f). Proteins are expressed in the whole lysate of aorta (*n *= 6) and heart (*n *= 6) from two groups of rats. Densitometric analysis was used to calculate normalized protein ratio (protein to β-actin). Data are expressed as mean ± SD. *P < 0.05, significantly different between HS-saline and HS-NaHS groups.

Compared to the HS-saline group, NaHS significantly decreased P-IκB and protein concentrations in the aorta (Figure 4a) and heart (Figure 4e) whereas NF-κB decreased only in the heart (Figure 4d). In addition, HS-NaHS treated rats showed a significant decrease in blotting for I-CAM in aorta (Figure 4c) but not in heart (*P *< 0.05) in comparison to the HS-saline group (Figure 4f).

### NaHS reduces I/R-induced oxidative stress

**Figure 5:**
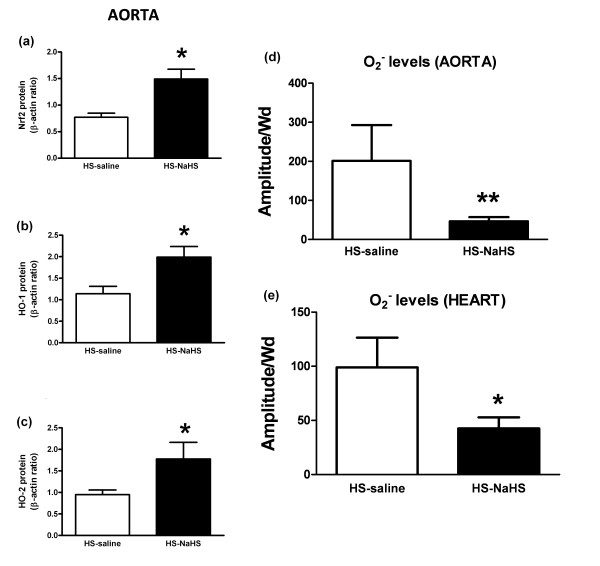
**Effects of NaHS on antioxidant pathway**. **(a, b, c) **Western blots revealing in aorta Nrf2 (a), HO-1 (b) and HO-2 (c) in the whole lysate of aortas (*n *= 6). **(d, e) **Quantification of the amplitude of O_2_^-^-Fe(DETC)_2 _signal in unit/weight (mg of the dried sample Amplitude/Wd, *n *= 10) in the aorta (d) and heart (e) of the two groups of rats. Data are expressed as mean ± SD. *P < 0.05 and **P < 0.01, significantly different between HS-saline and HS-NaHS groups.

Compared to the HS-saline group, Nrf2 was increased in aorta (*P *< 0.05) (Figure 5a) concomitant with a subsequent increase in HO-1 and HO-2 expressions (Figure 5b, c). However, NaHS did not decrease Nrf2, HO-1 and HO-2 (data not shown) in heart of the HS-NaHS group. Finally, compared to the HS-saline group, NaHS limited O_2_^- ^release in both tissues (*P *< 0.05) (Figure 5d, e).

## Discussion

In the present study, we report the beneficial effects of NaHS as an H_2_S donor, prior to retransfusion, in a rodent model of controlled hemorrhage. The key findings were that a single *i.v*. NaHS bolus immediately before retransfusion of shed blood (i) limited the I/R induced-decrease in MAP and (ii) was associated with reduced inflammatory and oxidative stress responses.

Although H_2_S is usually considered as an endogenous vasodilatator, this effect nevertheless remains a matter of debate. At low concentrations (10 to 100 μM H_2_S), Ali *et al. *[15] found a vasoconstrictor effect of H_2_S on rodent aorta, whereas Dombkovski [20] reported that H_2_S was responsible for either vasodilatation or vasoconstriction, according to species and organ requirements. Furthermore, data reported in the literature are highly conflicting: indeed, Mok *et al. *[17] reported an increase in MAP in unresuscitated HS treated with H_2_S synthesis blockers (DL-propargylglycine and μ-cyanoalanine) whereas Morrison *et al. *[16], using an opposite experimental approach, reported beneficial effects of H_2_S on survival in rats submitted to lethal unresuscitated HS. In the present study, compared to the HS-saline group, a single *i.v*. bolus of NaHS produced a substantial increase in MAP in hemorrhagic rats. All rats were well oxygenated (PaO_2 _>100 mm Hg, data not shown), an observation that was not reported in the studies by Mok *et al. *[17] and Morrison *et al. *[16].

The absence of a detrimental effect on stroke volume has already been reported by others [11,21,22]. Herein, heart rate was not altered in either group while carotid blood flow was higher in the HS-NaHS group. Since blood flow was decreased in HS-saline, this would suggest a higher stroke volume in HS-NaHS treated rats, although this conclusion could be challenged since cardiac output was not directly measured in this study. Nevertheless, this result is in agreement with improved ejection fraction in a model of myocardial I/R injury [23].

In the present study, NaHS treatment limited the metabolic acidosis induced by I/R. Simon *et al. *[21] also reported similar metabolic effects in pigs. Whether this effect is due to reduced metabolic demand induced by the sulfide donor or to a direct effect on mitochondrial K^+^_ATP _channels remains speculative since metabolic rate was not measured.

It is well documented that cardiovascular dysfunction during I/R is partly linked to the activation of the NF-κB/Rel pathway. This mechanism has been demonstrated in recent investigations [24], allowing the expression of iNOS and subsequent overproduction of NO in cardiovascular tissues [25]. As reported by others [26], we show herein that NaHS induced an *in vivo *down-expression of iNOs, with subsequent decrease in NO overproduction.

The effects of H_2_S on inflammation are also a matter of contention [25,27,28]. In the present model, we report a predominant inflammatory modulation effect. Indeed, NaHS was found to limit cardiovascular NF-κB activation as well as decrease I-CAM expression in aorta. These results confirm *in vitro *experiments which demonstrated that NaHS as well as other H_2_S endogenous donors modulate leukocyte-mediated inflammation [25,29] by decreasing leukocyte adhesion and leukocyte infiltration [23] through activation of K^+^_ATP _channels [25].

In the present study, infusion of a NaHS bolus attenuated oxidative stress induced by I/R, as mirrored by a decreased release of O_2_^- ^in tissues. H_2_S is known to react with the four different reactive oxygen species [30-32]. Since increased ROS formation is implicated in lipid peroxidation and oxidation of thiol groups, H_2_S, by decreasing ROS overproduction, may in fact limit tissue damage. Our results show that O_2_^- ^production was decreased in both aorta and heart, suggesting a protective effect on cardiovascular tissues. These results are in agreement with the observations of Sivarajah *et al. *[33], who recently reported that the cardioprotective effects of NaHS in a model of I/R on isolated cardiomyocytes were related to antioxidative and anti-nitrosative properties.

Nrf2 could contribute to adaptive and cytoprotective responses to various cell damages [31,34]. Different antioxidant cellular pathways are associated with Nrf2 expression such as the heme oxygenase enzymes, HO-1 and HO-2. Indeed, Maines *et al. *[30] reported increased levels of HO-1 in I/R injuries; moreover, HO-1 was found to improve resistance to oxidative stress [32] and modulate inflammatory response, particularly in hemorrhagic shock [35]. HO-2, meanwhile, is found in almost all tissues and is known as a potential O_2 _sensor in addition to playing a role in the maintenance of vascular tone [32]. Conversely to aortic tissues, there were no changes in Nrf2, HO-1 or HO-2 in the heart samples. In the present experimental design, rats were anesthetized and warmed but not overheated for ethical reasons in accordance with our animal care regulatory agency. The metabolic rate was not measured. In the studies of Blackstone *et al. *[10,11] and Morisson *et al. *[16], animals were awake. The difference between the two experimental protocols does not exclude a metabolic effect in our experiments. However, since body temperature remained constant throughout the study period, the putative effect of hypothermia did not significantly contribute to the observed results, which are related to reduced inflammatory and oxidative stress pathways. Consequently, the beneficial effect of NaHS is unlikely the result of a hibernation-like metabolic state of "suspended animation" as reported previously [10,11,16,22]. The present observation, however, confirms other studies in which H_2_S donors NaHS and Na_2_S protected against ischemia reperfusion injury [23,33,36-41] and burn injury [29] independently of core temperature.

### Study limitations

The present study has several limitations. By design, in order to mimic a realistic emergency clinical situation, we used a single *i.v*. dose of NaHS. Indeed, given the potential harmful effects of H_2_S on cytochrome c and the lack of data pertaining to the ideal target dose in the literature, we chose to infuse a single bolus dose of H_2_S. Since a dose-response study was not performed, it is possible that we may have missed toxic or beneficial potential effects of the hydrogen sulfide donor.

Moreover, we did not assess the effects of NaHS on inflammation and oxidative stress in non hemorrhagic rats since the injection of a single dose of 0.2 mg/kg of NaHS did not alter mean arterial pressure or carotid blood flow. The absence of vascular effects in non hemorrhagic rats may be related to the low infused dose or to the opposite effects of NaHS on isolated arteries. NaHS has been reported to exert a contractile activity mediated by the inhibition of nitric oxide and endothelial-derived hyperpolarizing factor pathways as well as a relaxation through both K^+^_ATP _channel-dependent and -independent pathways. In addition, Kubo *et al. *[14] reported only a very brief and reversible decrease in MAP (100 seconds) after *i.v*. injection of NaHS at 28 μmol/kg, which is equal to 0.31 mg/kg, a value close to the dose used in the present study. One could speculate that the beneficial effects of NaHS are unveiled in I/R situations when iNOS is up-regulated.

## Conclusions

The present *in vivo *experimental study of I/R following resuscitated hemorrhagic shock in rats demonstrates that a single *i.v*. bolus of NaHS limited the decrease in MAP during early reperfusion and down-regulated NF-κB, iNOS and I-CAM expressions. These anti-inflammatory effects were associated with decreased NO and O_2_^- ^production. Such beneficial effects of H_2_S donors warrant further experimental studies.

## Key messages

• The results of this *in vivo *experimental study demonstrate that a single *i.v*. bolus of hydrogen sulfide (considered as the third gaseous transmitter) donor, NaHS, prevented ischemia reperfusion (I/R)-induced hemodynamic dysfunction in a model of controlled hemorrhage in rats.

• NaHS reduced NO production and I/R-dependent iNOS expression and improved metabolic dysfunction.

• NaHS down-regulated NF-κB, iNOS and I-CAM expressions in this model.

• NaHS reduced I/R-induced oxidative stress.

## Abbreviations

CBF: carotid blood flow; CO: carbon monoxide; eNOS: endothelial nitric oxide synthase; EPR: electron paramagnetic resonance; FeDETC: N, N D-Ethyldithiocarbamate and Fe^3^+ citrate complex HO-1: heme-oxygenase-1; HO-2: heme-oxygenase-2; HR: heart rate; HS: hemorrhagic shock; H_2_S: hydrogen sulfide; iNOS: inducible NOS; I/R: ischemia-reperfusion; *i.v*.: intravenous; MAP: mean arterial pressure; NaHS: sodium hydrosulfide; NO: nitric oxide; Nrf2: nuclear respiratory factor 2; O_2 _: superoxide anion; PI-κB: phosphorylated I-κB; PMSF: phenylmethylsulfonyl fluoride; ROS: radical oxygen species; SD: standard deviation.

## Competing interests

The authors declare that they have no competing interests.

## Authors' contributions

FG participated in the surgical procedure, in *in vitro *measurements and in the design of the protocol, and drafted the manuscript. MB carried out the Western blotting. MdlB and LF carried out the surgical procedure and *in vitro *measurements. OD participated in the laboratory investigations. AM, PC and DH helped to design the study. PR helped to design the study and to draft the manuscript. LL participated in *in vitro *measurements. PA designed the study, and coordinated and drafted the manuscript. FM participated in the design of the study, performed the statistical analysis and helped to draft the manuscript.
